# Radar-based inspiratory-to-expiratory time ratio estimation: a validation study

**DOI:** 10.1038/s41598-026-42517-9

**Published:** 2026-03-04

**Authors:** Thanh Trúc Trần, Marie Oesten, Stefan G. Griesshammer, Anke Malessa, Kilin Shi, Maria Heckel, Bjoern M. Eskofier, Alexander Koelpin, Christoph Ostgathe, Tobias Steigleder

**Affiliations:** 1 Department Artificial Intelligence in Biomedical Engineering (AIBE), Friedrich-Alexander-Universität Erlangen-Nürnberg (FAU), Erlangen, Germany; 2 Department for Palliative Care, University Hospital Erlangen, Friedrich- Alexander-Universität Erlangen-Nürnberg (FAU), Erlangen, Germany; 3 Institute for Electronics Engineering, Friedrich-Alexander-Universität Erlangen-Nürnberg (FAU), Erlangen, Germany; 4 Chair of AI-supported Therapy Decisions, Institute for Medical Information Processing, Biometry, and Epidemiology, LMU München, Munich, Germany; 5 Translational Digital Health Group, Institute of AI for Health, German Research Center for Environmental Health, Helmholtz Zentrum München, Neuherberg, Germany; 6 Munich Center for Machine Learning (MCML), Munich, Germany; 7 Institute for High Frequency Technology, Technische Universität Hamburg (TUHH), Hamburg, Germany

**Keywords:** Radar-based vital sign monitoring, Biomarkers, Respiration, Inspiratory-to-expiratory time ratio, Diseases, Health care, Medical research, Signs and symptoms

## Abstract

Respiration is a key indicator of health and wellbeing, with metrics such as respiratory rate (RR), inspiratory time (TI), expiratory time (TE), and the inspiratory-to-expiratory time (I:E) ratio offering insights into conditions ranging from acute life-threatening and chronic diseases to symptom management. While traditional methods already measure these parameters with high accuracy, they still require contact-based sensors, limiting their practicality for continuous monitoring. This study evaluates radar as a non-contact alternative by validating multiple radar-derived respiratory metrics against impedance pneumography measurements in 30 healthy volunteers at rest. Synchronous recordings from both modalities were analysed to assess agreement across methods using descriptive statistics, scatter plots, modified Bland-Altman plots, and equivalence testing (TI: ±0.3 s, TE: ±0.3 s, RR: ±2 brpm, I:E ratio: ±0.2). Equivalence testing indicated high correlation (*p* ≤ 0.001***) across all metrics, with 81.8% (TI), 77.6% (TE), 97.2% (RR), and 85.7% (I:E ratio) of values within predefined bounds. These findings highlight radar’s potential for continuous respiratory monitoring, particularly in medical fields where minimizing patient burden is essential as in palliative, post anaesthesia, and intensive care settings.

**Supplementary Information:**

The online version contains supplementary material available at 10.1038/s41598-026-42517-9.

## Introduction

Respiration is a key vital sign that provides insights into a person’s health and is therefore routinely assessed by healthcare professionals (HCPs) for patient monitoring and disease management. The respiration cycle consists of two phase – inspiration and expiration – which enable the exchange of oxygen and carbon dioxide^1^.

Abnormal respiratory patterns can indicate a range of acute and chronic conditions, from serious systemic infections^2,3^ and chronic respiratory diseases^4–7^ to cardiovascular illnesses like heart failure^8,9^. These conditions often lead to compensatory changes in breathing regulation including altered breathing frequency and disturbed inspiratory and expiratory dynamic, which can result in critical impairments of gas exchange if left unmanaged^10^. Continuous and accurate respiratory monitoring is therefore essential for early detection and timely intervention^11^.

Key respiratory metrics include respiratory rate (RR), inspiratory time (TI), expiratory time (TE), and the inspiratory-to-expiratory time (I:E) ratio, which together provide complementary information on respiratory function. RR reflects the overall breathing frequency and is widely used for detecting acute respiratory distress^12^ or metabolic imbalances. However, RR alone captures only the global periodicity of breathing and does not reflect the temporal structure of individual respiratory cycles. In contrast, TI, TE, and particularly the I:E ratio characterize the relative lengths of the inspiratory and expiratory phases and are sensitive to changes in airway resistance and lung compliance^13^. Consequently, the I:E ratio plays a central role in mechanical ventilation for monitoring and adjusting ventilatory support^14,15^.

Despite their clinical relevance, phase-based respiratory parameters are more challenging to assess than RR, as they require accurate detection of respiratory phase transitions and are more susceptible to noise, motion artifacts, and irregular breathing patterns. This increased methodological complexity may partly explain why most contactless respiratory monitoring studies to date have primarily focused on RR, while systematic validation of TI, TE, and I:E ratio remains limited^16^.

Various traditional methods such as spirometry^17–19^, respiratory inductive plethysmography^20,21^, and impedance pneumography^22–24^ are well established for measuring respiration with high accuracy. While these methods provide precise measurements, they rely on unwieldy, contact-based equipment, which can be burdensome for patients, restrict motion and autonomy, and rely on specially trained HCPs to apply and to maintain the system. Thus, these diagnostic tools are impractical for quick and efficient use in emergency room settings and are not suitable for long-term continuous monitoring^25,26^.

To address these limitations, various contactless technologies have been explored like camera-based systems^27,28^ and thermal imaging^29,30^. However, these approaches entail several disadvantages, such as dependence on specific lighting conditions, line-of-sight constraints, controlled ambient temperatures, and potential privacy and data protection issues. In contrast, radar-based sensing^31–33^ has emerged as a promising alternative, being independent of those restrictions. Another key advantage is its operational simplicity, as no additional personnel is required to initiate or conduct measurements. Furthermore, radar waves can penetrate materials such as mattresses, bedding, and clothing, allowing for seamless integration into patient environments by placing a radar system underneath the bed^34^. Radar systems can also simultaneously measure other vital parameters, such as heart rate^35,36^, offering a comprehensive physiological assessment by a single device.

Most radar-based respiratory studies to date focused primarily on measuring RR^37^, typically derived from dominant spectral components or peak-to-peak intervals of the respiratory signal. Although RR is a fundamental parameter, it only captures part of the respiratory dynamics. Parameters such as TI, TE and I:E ratio provide deeper insights into respiratory effort, airflow limitations, and abnormal breathing mechanics, which can be relevant for diagnosing and managing various conditions. Despite the potential of radar technology to extract these metrics, systematic validation studies remain lacking.

This study addresses this gap by providing a systematic validation of radar-based TI, TE, and the I:E ratio alongside RR against impedance pneumography, the established reference, to ensure the precision and robustness of the proposed approach. In doing so, this work contributes a comprehensive evaluation of radar-based extraction of multiple respiratory metrics beyond RR, assessing their accuracy and robustness under varying respiratory conditions. Furthermore, the study demonstrates the feasibility of using radar sensing for detailed, contactless respiratory monitoring, thereby laying the foundation for future clinical applications.

## Materials and methods

### Ethics approval

All research were performed in accordance with the relevant guidelines and regulations and in accordance with the Declaration of Helsinki. The study protocol was approved by the local ethics committee of the Friedrich-Alexander-Universität Erlangen-Nürnberg (No. 85_15B). Written informed consent was obtained from all participants prior to inclusion in the study.

The dataset analysed in the present work was previously published by Schellenberger et al.^38^ and is publicly available at figshare^39^; no additional data were collected for the purposes of this study.

### Overview of the dataset

The dataset comprises 24 h of synchronized data from a radar system and a reference device, collected from 30 healthy participant. The study is listed in the central study register of the Bavarian Cancer Research Center (BZKF) under the acronym GUARDIAN^40^.

While the original dataset includes measurements from various induced scenarios, such as the Valsalva manoeuvre and tilt table test, this work focuses exclusively on data from the resting scenario. This restriction was chosen to establish a controlled baseline condition for validating the radar-derived respiratory metrics under stable, low-motion circumstances. Analysing resting data minimizes confounding effects from movement, posture changes, or voluntary breathing alterations, thereby enabling a more precise comparison between radar and reference signals.

During the resting scenario, participants lay relaxed on the horizontal tilt table, breathing calmly for at least 10 min while avoiding large movements. This resulted in approximately 5.3 h of recorded data available for analysis in this study.

The continuous wave (CW) radar system, based on Six-Port technology, is described in more detail below. As a reference system, the CNSystems Task Force^®^ Monitor (TFM) simultaneously recorded electrocardiogram (ECG), thoracic impedance, impedance cardiogram (ICG), and non-invasive continuous blood pressure.

### Radar system

The radar system^38^ used for data collection is a portable CW radar operating at 24 GHz within the industrial, scientific, and medical (ISM) frequency band and is mounted above the tilt table^38^. To enhance signal quality, the system employs a bistatic antenna design with an inclination angle of ± 10° and a focal point at 40 cm. A built-in class 1 positioning laser, located between the transmitting (TX) and receiving (RX) antennas, assists in alignment by projecting onto the participant’s upper body.

The system captures body motion by measuring phase changes (Δφ) between transmitted and received signals. The phase angle is derived from the in-phase (I) and quadrature (Q) components of the radar raw signal through arctangent demodulation^41^ as:

1 $$\begin{array}{c}\varDelta\varphi=atan2\left(\frac{Q}{I}\right)\end{array}$$

The corresponding displacement (Δx) from a reference point is then computed from the measured phase difference, given the known wavelength λ of the TX signal:

2 $$\begin{array}{c}\varDelta x=\frac{\varDelta\varphi}{2\pi}\cdot\frac{\lambda}{2}\end{array}$$

In practice, however, before demodulation can be applied, ellipse reconstruction is required to compensate for hardware non-idealities such as channel imbalances and DC offsets. The detailed procedures are described in the section “Data preprocessing”.

The Six-Port structure allows for high phase resolution, and the I/Q signals are digitized at 2000 Hz using a 24-bit analog-to-digital converter (ADC), then stored in a microcontroller and transmitted via Ethernet for further analysis. To enable synchronization with the reference system, the microcontroller also generates a binary sequence, which is sampled alongside the radar data and fed into the external input of the TFM.

### Reference system

The reference system used in this study is the TFM 3040i (CNSystems Medizintechnik GmbH, Austria), a commercially available and clinically established impedance cardiography system for non-invasive cardiovascular monitoring^42^. The TFM records multiple physiological signals, including thoracic impedance and ICG, using surface electrodes placed on the upper body.

In this setup, a small alternating current is applied across the thorax via four electrodes. The system measures the resulting thoracic impedance (Z) and its time derivative (–dZ/dt), latter representing the ICG. The raw ICG signal reflects rapid impedance changes associated with cardiac activity, whereas the low-frequency component of the impedance signal corresponds to respiratory-induced variations and is therefore used for impedance pneumography^43,44^. The exported data include the ICG signal sampled at 500 Hz and the impedance signal sampled at 50 Hz, which serves as the respiratory reference in this study. As noted by the authors of the dataset^38^, the impedance signal was upsampled to 100 Hz to enable synchronization between the TFM and radar systems through an external input channel recording a binary trigger sequence from the radar alongside the physiological signals.

In this study, the thoracic impedance signal was used to derive respiratory activity, which was processed offline using a custom signal processing pipeline. No proprietary respiratory analysis provided by the manufacturer was used.

### Data preprocessing

**Fig. 1:**
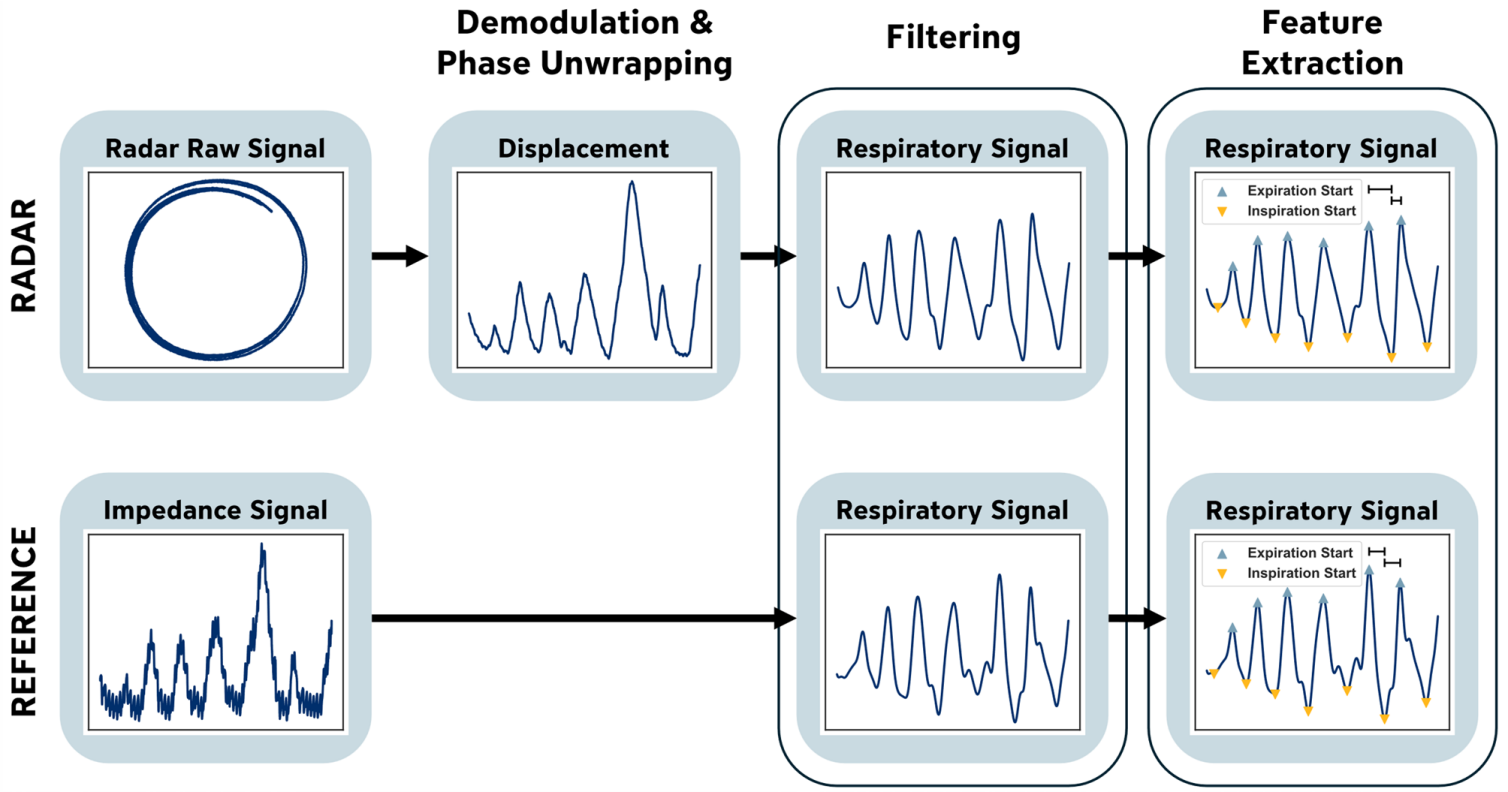
Overview of the key data preprocessing elements of the presented approach, including demodulation, phase unwrapping, and filtering to extract respiratory signals, followed by feature extraction.

Figure 1 provides an overview of the three key preprocessing steps, each of which is described in detail below. All processing was performed using *Python* (version 3.10.11). Since the dataset used in this study already contains synchronized radar and reference signals, no additional temporal alignment is required. Additionally, as the analysis focuses on time-resolved respiratory metrics derived from the timing of local extrema, resampling was intentionally avoided. Preserving the original sampling rates of the radar and reference system prevents potential distortions of phase timing that could arise from interpolation, which is critical for accurate estimation of phase-based respiratory metrics.

Radar displacement was extracted from the I/Q signals using ellipse reconstruction followed by arctangent demodulation^34,45^. Ellipse reconstruction compensates for hardware-induced I/Q imbalances, ensuring that the derived phase predominantly reflects chest displacement rather than system artifacts. The instantaneous phase was then obtained via arctangent demodulation (Eq. 1, see “Radar System”). Since the arctangent output is inherently wrapped between -π and π, phase unwrapping (Eq. 2, see “Radar System”) is then applied to obtain a continuous phase signal representing chest displacement in millimetres. Finally, to isolate the radar-based respiratory signal, a fourth-order Butterworth bandpass filter is applied in the range of 0.07–0.4 Hz, which corresponds to RRs roughly between 4 and 24 breaths per minute (brpm). This range includes the normal physiological RR in healthy adults (12–20 brpm) while allowing for moderate deviations due to variability or measurement uncertainty.

**Fig. 2:**
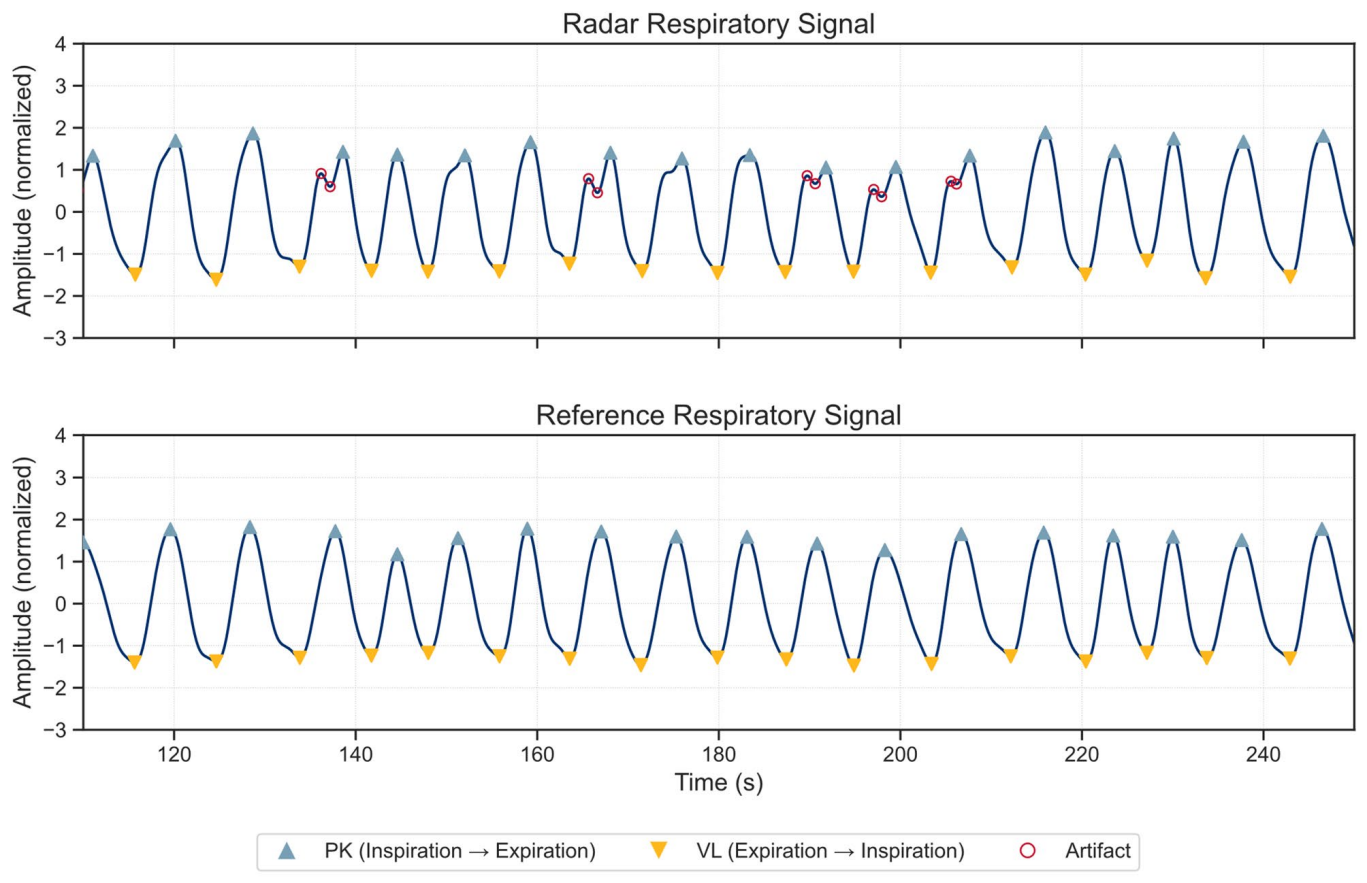
Synchronized and normalized breathing signals derived from radar and reference sensors. Peaks (PKs) and valleys (VLs) indicate transitions from inspiration to expiration and from expiration to inspiration, respectively. Artifacts mark local extrema that are not associated with actual respiratory phase transitions.

The same bandpass filter is used on the reference signal derived from impedance data. Since the radar displacement signal is measured in millimetres and the reference impedance signal in Ohms, both filtered signals are then normalized using a standard scaler to facilitate comparison across modalities. Figure 2 presents an example of synchronized and normalized respiratory signals from both the radar and reference systems.

The first step of the feature extraction consisted of detecting local extrema in the radar and reference respiratory signals^46^. Local maxima (peaks, PKs) and minima (valleys, VLs) were defined as points where the first derivative of the signal is zero and the second derivative is negative or positive, respectively. A complete respiratory cycle was defined as a sequence of inspiration (VL → PK) followed by expiration (PK → VL). To suppress artifact-related extrema, physiologically motivated constraints on amplitude prominence, temporal distance, and peak width were applied during extremum detection. These constraints were chosen based on expected resting breathing characteristics in healthy adults and refined empirically to ensure robust detection across subjects.

Because PKs and VLs were detected independently, their temporal order was constrained to the expected VL → PK → VL pattern. In cases of consecutive extrema of the same type, only the first PK and the last VL were retained to preserve physiologically plausible inspiration-expiration asymmetry as TE exceeds TI in normal breathing. These concepts are illustrated in Fig. 2, which shows examples of PKs and VLs detected separately for the radar and reference respiratory signals. Detailed explanation and parameter settings of local extrema detection and artifact handling are provided in the Supplementary Methods section.

Once individual respiratory cycles are identified, cycles with a total duration exceeding 10 s (corresponding to < 6 brpm) are classified as outliers and excluded from further analysis as they are considered physiologically unrealistic for healthy volunteers.

Respiratory metrics, specifically RR, TI, TE, and I:E ratio, are computed using a sliding window approach. A window size of 2 min was chosen to balance the need for sufficient respiratory cycles to compute stable average metrics while retaining temporal resolution for monitoring short-term changes. A step size of 30 s introduces overlap between consecutive windows. This approach is applied to both the radar and reference signals.

Within each window, only complete cycles, defined as those where both the starting and ending VLs fall within the 2-minute window, are considered. For each respiratory cycle within a window, TI is calculated as the time from VL to PK, and TE as the time from PK to the subsequent VL.

To minimize the distortion caused by occasional extreme values in the sliding window without discarding information from the majority of cycles, the cycles with the smallest and largest TI and TE are excluded before computing the mean metrics (at minimum two and at maximum four cycles are excluded). While the median is robust to outliers, it represents only the central value of the distribution and can underrepresent subtle shifts in respiratory timing across multiple cycles. Excluding only the extremes allows us to retain sensitivity to genuine variations while still reducing the influence of outliers.

After outlier exclusion, mean TI and mean TE are calculated from the remaining cycles. Mean RR is then determined by taking the inverse of the sum of TI and TE for each cycle, followed by calculating the mean of these values. Similarly, mean I:E ratio is calculated by taking the quotient of TI and TE for each cycle, then computing the mean of these ratios. Each interval is then represented solely by these mean metrics, making the individual cycle metrics irrelevant for subsequent analysis.

### Statistical analysis

Statistical analyses were performed using *Python* (version 3.10.11), with *scipy* (version 1.15.2) for statistical computations and *matplotlib* (version 3.10.1) for visualization. To evaluate the agreement between the proposed method and the reference, we conducted a concordance analysis following Müller and Büttner^47^, incorporating both visual and statistical methods.

First, for each of the four respiratory metrics of interest (TI, TE, RR, and I:E ratio), scatter plots were created, displaying radar-based mean values against the corresponding reference mean values. The scatter plots include the regression line, which can be compared to the line of identity to assess the agreement between radar-based and reference measurements. Additionally, modified Bland-Altman plots were generated for each case, as described in^48^. These plots depict the mean difference (radar vs. reference) on the y-axis against the reference value on the x-axis, with limits of agreement set within a 95% confidence interval.

Following this, a two one-sided test (TOST) was performed according to Schuirmann^49^ to assess the equivalence of the measurement methods. The equivalence bounds in Table 1 were defined based on clinically acceptable deviations for each metric, reflecting differences that would not meaningfully affect respiratory assessment. Since no standardized limits for radar-based respiratory monitoring exist, the chosen bounds represent conservative estimates derived from typical physiological variability and expected measurement precision.

**Table 1:** Equivalence boundaries for TOST across respiratory metrics.

Metric	Lower border $-{\boldsymbol{\Delta}}_{\boldsymbol{L}}$	Upper border ${\boldsymbol{\Delta}}_{\boldsymbol{U}}$
RR (brpm)	− 2	+ 2
TI (s)	− 0.3	+ 0.3
TE (s)	− 0.3	+ 0.3
I:E Ratio (–)	− 0.2	+ 0.2

Thus, for each metric, the null hypothesis tested was:

$$\begin{array}{*{20}c} {Metric_{\text{mean radar}} - Metric_{\text{mean reference}} \le - \Delta _{L} } \\ {or} \\ {Metric_{\text{mean radar}} - Metric_{\text{mean reference}} \ge \Delta _{U} } \\ \end{array}$$

If the null hypothesis is accepted, the measurements are not equivalent. If the null hypothesis is rejected, the alternative hypothesis is accepted:

$$-{{\Delta}}_{L}<{Metric}_\text{mean radar}-{Metric}_\text{mean reference}<{{\Delta}}_{U}$$

This indicates that the two measurement methods are considered equivalent. A significance level of α = 0.05 was used for the Type I error, i.e. to conclude equivalence the 90% confidence interval (CI) must lie entirely within the equivalence bounds.

## Results

### Participants and data evaluation

*N* = 30 participants were included in this study, with an almost even gender distribution (16 female). The average age was 30.7 ± 9.9 years (range: 21–61 years), with a mean weight of 72.2 ± 14.0 kg and an average BMI of 23.2 ± 3.3 kg/m².

In total, 5.3 h of resting-state data were recorded, containing 3,466 full respiratory cycles in the radar signals and 3,474 in the reference signals. However, after excluding outlier cycles with durations over 10 s, only 3,284 radar and 3,300 reference respiratory cycles are considered for further analysis. Using a sliding window approach with a 2-minute window and a 30-second step size, 532 intervals were generated for comparison of the measurement methods.

### Descriptive analysis

**Fig. 3:**
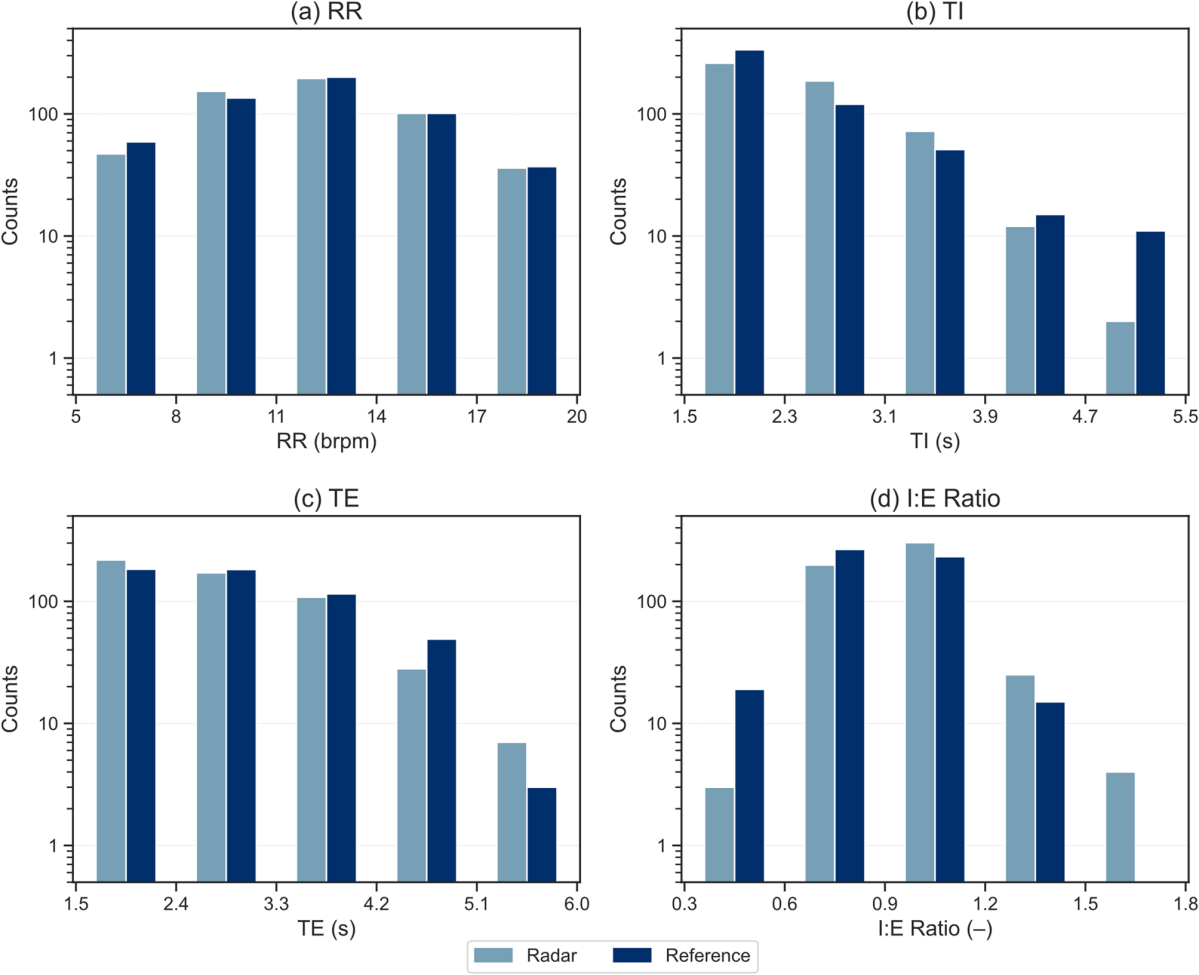
Distribution of respiratory metrics for radar and reference measurements. **a** Respiratory rate (RR); **b** inspiratory time (TI); **c** expiratory time (TE); **d** inspiratory-to-expiratory time (I:E) ratio.

Descriptive statistics for the radar and reference respiratory metrics are presented in Table 2. Additionally, Fig. 3 shows histograms illustrating the distribution of the respiratory metrics for radar-based and reference measurements. For the RR histogram in Fig. 3a, the distribution appears to be roughly symmetrical, centred around 11–14 brpm, which is comparable to the lower end of the expected resting range in healthy adults.

Histograms in Fig. 3b and c show that the distributions for TI and TE are positively skewed, with a higher concentration of values on the left side (lower values), reflecting a predominance of shorter phase durations with fewer prolonged events. For both measurement modalities, TI was shorter on average than TE, consistent with expected physiological resting breathing patterns. A similar relationship was observed for the maximum TI and TE values. Minimum TI and TE values were comparable between radar and reference measurements.

The I:E ratio histogram in Fig. 3d appears similar to the RR histogram in that the distribution is approximately symmetrical, with most values centred around 0.9 for both modalities. However, a noticeable difference in the I:E ratio plot is the presence of higher-value outliers (values > 1.5), which occurred only in the radar-derived measurements.

**Table 2:** Descriptive statistics of respiratory metrics of radar and reference using 532 intervals.

		Minimum	Maximum	Mean	Standard Error σₘ	Standard Deviation σ
RR (brpm)	Radar	6.20	18.70	12.13	0.13	2.90
	Reference	6.33	18.72	12.08	0.13	3.02
TI (s)	Radar	1.61	5.26	2.46	0.03	0.60
	Reference	1.60	5.15	2.41	0.03	0.69
TE (s)	Radar	1.59	5.56	2.79	0.04	0.84
	Reference	1.59	5.50	2.91	0.04	0.89
I:E Ratio (–)	Radar	0.53	1.54	0.93	0.01	0.15
	Reference	0.51	1.45	0.87	0.01	0.15

### Concordance analysis

To evaluate the agreement between radar-derived and reference respiratory measurements, a concordance analysis was performed for each respiratory metric, respectively. Mean values of each metric were computed for all 532 analysed time intervals across subjects.

**Fig. 4:**
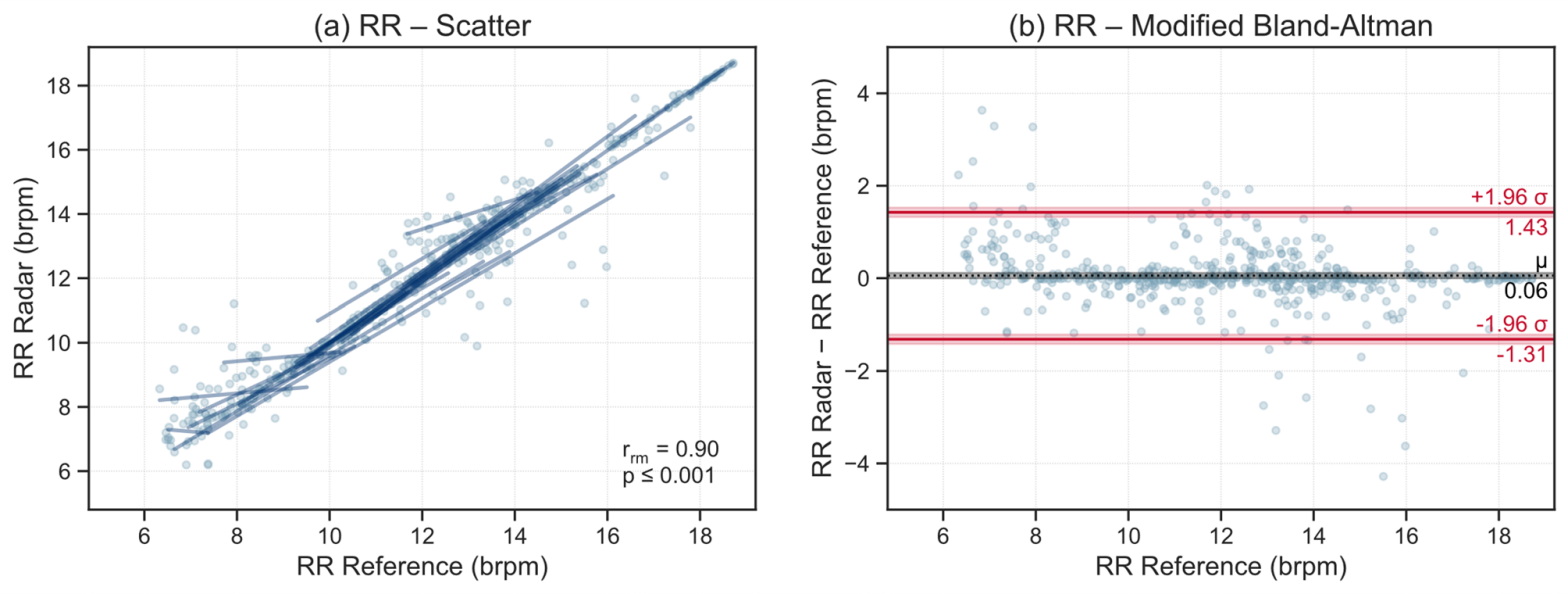
**a** Scatter plot and **b** modified Bland-Altman plot for radar- and reference-derived respiratory rates (RRs). Scatter plots show within-subject repeated-measures correlations r_rm_, and modified Bland-Altman plots display mean bias µ and limits of agreement (± 1.96σ).

**Fig. 5:**
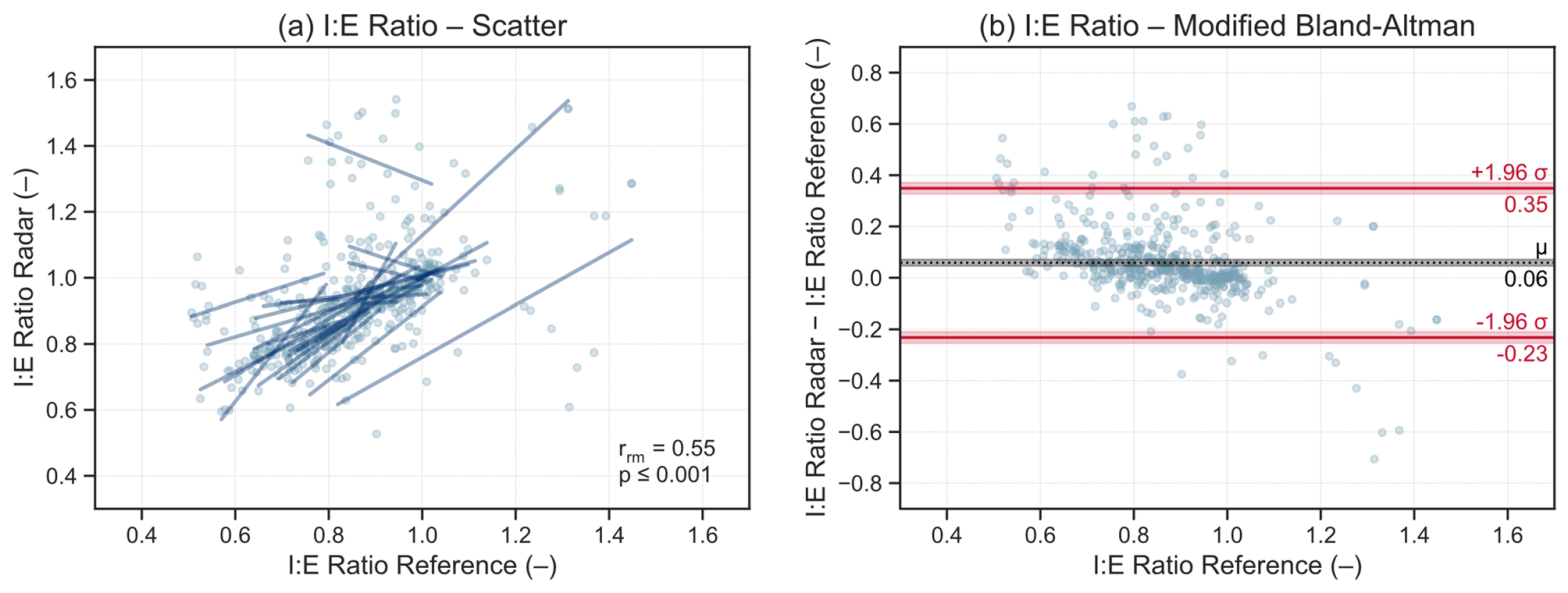
**a** Scatter plot and **b** modified Bland-Altman plot for radar- and reference-derived inspiratory-to-expiratory time (I:E) ratios. Scatter plots show within-subject repeated-measures correlations r_rm_, and modified Bland-Altman plots display mean bias µ and limits of agreement (± 1.96σ).

Results for RR and the I:E ratio, which represent the primary outcome measures of this study, are presented in Figs. 4 and 5. Corresponding analyses for TI and TE are provided in the Supplementary Fig. S1 and S2. The scatter plots illustrate the relationship between radar and reference measurements, showing individual regression lines per subject to visualize within-subject trends. Additionally, within-subject repeated-measures correlation coefficients r_rm_ and their associated *p*-values are reported. The modified Bland-Altman plots depict the mean difference µ between measurement methods and the limits of agreement µ ± 1.96σ, where σ denotes the standard deviation of the differences. Shaded confidence bands around these lines indicate the uncertainty of the estimated bias and limits of agreement.

Figure 4a and b show the comparison of RR between radar and reference measurements. Most subject-wise regression lines closely follow the line of identity, indicating strong agreement between methods (r_rm_ = 0.90, *p* ≤ 0.001). The RR difference between methods lies within the ± 2 brpm equivalence bounds for 517 of 532 intervals (97.2%). As shown in the modified Bland-Altman plot (Fig. 4b), 503 of 532 differences (94.6%) fall within the limits of agreement (µ ± 1.96σ = 0.06 ± 1.37 brpm). The narrow limits and the near-zero mean bias demonstrate high consistency and minimal systematic error between radar and reference RR measurements.

Supplementary Fig. S1a and S1b depict the comparison for TI. The repeated-measures correlation indicates moderate agreement (r_rm_ = 0.70, *p* ≤ 0.001). While most subject-wise regression lines follow the line of identity (i.e., perfect correspondence between radar and reference values), four participants deviate notably: some exhibit inverse relationships (negative slopes), whereas others show near-horizontal regression lines, indicating little to no variation in radar-derived TI despite pronounced changes in the reference measurements. These outliers are especially prominent at higher reference TI values and substantially reduce the overall correlation. The modified Bland-Altman plot reveals an overall mean TI difference of µ = 0.05 s, with 435 of 532 intervals (81.8%) falling within the ± 0.3 s equivalence bounds and with 503 of 532 intervals (94.6%) falling within the limits of agreement (µ ± 1.96σ = 0.05 ± 0.58 s).

Supplementary Fig. S2a and S2b compare TE values between radar and reference. The repeated-measures correlation was moderately strong (r_rm_ = 0.80, *p* ≤ 0.001), partly due to one subject exhibiting an inverse trend. Differences lie within the ± 0.3 s equivalence bounds for 413 of 532 cases (77.6%), while 493 intervals (92.7%) fall within the limits of agreement (µ ± 1.96σ = −0.12 ± 0.74 s). The small negative bias indicates a slight underestimation of TE by radar, with most deviations occurring for a small number of subjects and at higher TE values.

Figure 5a and b illustrate the comparison of the I:E ratio, which showed the weakest agreement among all metrics (r_rm_ = 0.55, *p* ≤ 0.001). Visual inspection reveals that the overall correlation is strongly influenced by a small number of participants whose subject-wise regression patterns deviate notably from the majority. The mean difference between methods was µ = 0.06, with 456 of 532 intervals (85.7%) within the ± 0.2 equivalence bounds and 496 (93.2%) within the limits of agreement (µ ± 1.96σ = 0.06 ± 0.29).

### Test for equivalence

The results of the equivalence test for the data obtained via radar and reference measurements are summarized in Tables 3 and 4. The TOST results (Table 4) indicate statistically significant results across all metrics (*p* ≤ 0.001). The raw effect sizes presented in Table 4 confirm this result, as the 90% CI lower and upper bounds of the raw effect lie within the equivalence bounds -${{\Delta}}_{L}$ and ${{\Delta}}_{U}$ of the TOST. This indicates that the null hypothesis of non-equivalence is rejected, thereby demonstrating equivalence for all four respiratory metrics.

In addition to statistical significance, the direction of the effects across both raw and standardized measures should be noted. Negative values for RR and TE indicate that radar estimates are generally lower than those of the reference system, while TI and I:E ratio tend to be higher.

The equivalence testing results support the findings of the concordance analysis. Together with the small raw effect sizes (Table 4) and the near-zero mean biases observed in the Bland-Altman analysis (Figs. 4 and 5), the TOST results (Table 3) confirm equivalence between radar and reference measurements for all metrics within the predefined equivalence margins.

**Table 3:** Comparison of measurement methods: equivalence testing of using TOST.

Metric	Test Bound	t	df	*p*	Equivalence Margin
RR	TOST lower	67.81	531	≤ 0.001***	± 2 brpm
	TOST upper	−64.11	531	≤ 0.001***	
TI	TOST lower	27.75	531	≤ 0.001***	± 0.3 s
	TOST upper	−19.27	531	≤ 0.001***	
TE	TOST lower	11.24	531	≤ 0.001***	± 0.3 s
	TOST upper	−25.61	531	≤ 0.001***	
I:E Ratio	TOST lower	40.21	531	≤ 0.001***	± 0.2
	TOST upper	−21.99	531	≤ 0.001***	

**Table 4:** Comparison of measurement methods: raw effect sizes and Cohen’s d_z_.

Metric	Measure	Value	90% CI Lower	90% CI Upper
RR	Raw Effect Size (brpm)	0.06	0.01	0.11
	Cohen’s d_z_	0.08	0.01	0.15
TI	Raw Effect Size (s)	0.05	0.03	0.08
	Cohen’s d_z_	0.18	0.11	0.26
TE	Raw Effect Size (s)	−0.12	−0.14	−0.09
	Cohen’s d_z_	−0.31	−0.38	−0.24
I:E Ratio	Raw Effect Size	0.06	0.05	0.07
	Cohen’s d_z_	0.39	0.32	0.47

## Discussion

This study aimed to validate radar as a reliable method for measuring respiratory signals in a resting scenario by comparing it with impedance pneumography, the chosen reference. The results demonstrate that radar technology can robustly capture respiratory dynamics under the investigated conditions, with metric-specific differences in agreement reflecting both physiological and methodological factors.

### Agreement across respiratory metrics

Statistical analyses confirmed the equivalence of radar- and reference-derived measurements. Both the modified Bland-Altman and equivalence (TOST) analyses indicated that radar-based values fell within medically acceptable limits, with small effect sizes supporting practical agreement. These findings underscore the radar’s potential as a non-invasive alternative for continuous respiratory monitoring.

Radar-derived RR showed strong concordance with the reference (r_rm_ = 0.90) and negligible bias. The narrow limits of agreement, together with the high proportion of observations falling within clinically predefined equivalence bounds, indicate that radar can estimate RR with accuracy comparable to established reference methods. This finding aligns with previous contactless respiration studies^37^, reaffirming that RR is the most robust radar-based parameter.

For TI and TE, correlations were moderately strong (r_rm_ = 0.70 and 0.80, respectively). Most values lay within the predefined equivalence ranges, but a small systematic deviation was observed: radar tended to underestimate longer TI and TE values, particularly at higher values of the reference measurement. This pattern suggests a duration-dependent bias rather than a phase-specific limitation. Physiologically, longer phase durations are typically associated with slower, lower-amplitude thoracic motion, as observed during relaxed breathing. Under these conditions, reduced motion dynamics may diminish the signal-to-noise ratio of the radar displacement signal, causing detected phase boundaries to shift toward regions of higher local signal dynamics rather than the true physiological extrema. This shift can result in systematically shorter estimated inspiratory and expiratory durations. Despite this, the mean differences for TI (0.05 s) and TE (− 0.12 s) remained small, suggesting limited clinical relevance.

The weaker agreement observed for the I:E ratio (r_rm_ = 0.55) appears to be driven predominantly by a small subset of participants rather than a uniform degradation in performance across the cohort. This subject-specific variability suggests that individual breathing patterns play a critical role in the robustness of ratio-based metrics. From a methodological perspective, inspection of these cases did not indicate a systematic failure of the peak and valley detection algorithm itself, but rather highlighted conditions in which the underlying radar signal exhibited reduced amplitude or less distinct extrema. In particular, participants exhibiting prolonged or irregular respiratory phases may produce lower-amplitude or less distinct thoracic motion, which can impair phase boundary detection and disproportionately affect derived parameters such as the I:E ratio. Consequently, even subtle timing deviations in TI or TE can be amplified at the subject level, leading to substantial inter-individual variability in I:E ratio agreement.

### Limitations and future works

Accurately measuring respiratory metrics requires precise identification of the start and end of inspiration and expiration phases, which are determined by local PKs and VLs in the respiratory signal. Incorrect identification not only distorts the computed I:E ratio for that cycle but also affects subsequent respiratory cycles.

Artifacts may arise from random body movement^50^, cardiac interference^51^, or irregular breathing patterns such as coughing, vocalizing or swallowing^52,53^. To mitigate such effects, we applied prominence thresholds for PKs and VLs, based on the standard deviation of their amplitudes within one measurement, and imposed minimum distance and width constraints consistent with realistic respiratory cycles. This ensured temporal plausibility and reduced false detections. Additionally, to maintain physiological consistency (VL → PK → VL), only the first PK and last VL were retained when multiple extrema occurred in succession. While this approach enforces a consistent cycle structure and accounts for the typical asymmetry between inspiratory and expiratory phases, it assumes a regular breathing pattern. Thus, it may not generalize to highly irregular or pathological respiration. Future work should explore more advanced, data-driven methods that are less constrained by these assumptions and better able to adapt to a wider range of breathing patterns^54^.

Differences in sampling frequency may also have influenced results. The radar signal was sampled at 2000 Hz, while the impedance pneumography signal was recorded at 100 Hz (upsampled from 50 Hz). While synchronization minimizes alignment errors, differing temporal resolutions could still introduce minor timing discrepancies, particularly in short inspiratory or expiratory phases. While these shifts minimally affect RR, which depends on the total cycle duration, they can accumulate in the I:E ratio, which relies on individual TI and TE durations.

The evaluation was limited to a resting-state scenario with relatively short recordings (approximately 10 min per subject) in a controlled environment. Future research should examine a broader range of breathing patterns and include conditions that induce variability, such as physical activity, emotional stress, or clinical interventions. Such data would better reflect real-world conditions and allow assessment of radar performance under more dynamic scenarios. In addition, longer recordings are required to evaluate long-term stability and robustness, particularly for continuous monitoring applications.

Finally, ongoing advances in radar hardware and signal processing (i.e., real-time implementation, machine learning-based event detection) are expected to further improve metric precision and enhance applicability in both clinical and home settings.

### Clinical implications and potential applications

The demonstrated agreement between radar- and reference-derived respiratory metrics under resting conditions suggests that radar-based monitoring may be suitable for a range of clinical applications requiring continuous, unobtrusive respiratory assessment, particularly for capturing diagnostically and prognostically relevant information related to the I:E ratio. In sleep monitoring, radar-based systems may help detect sleep apnoea by identifying irregular respiratory cycles without physical contact. In end-of-life care, radar-based monitoring could support clinical decision-making by providing continuous, unobtrusive respiratory assessment, helping clinicians detect signs of discomfort or the need for symptom management while maintaining a calm and dignified environment for the patient. In the post anaesthetic care unit (PACU), continuous radar-based monitoring could enable earlier detection of postoperative respiratory complications, such as opioid-induced respiratory depression^55^. Integrated systems positioned in or below the patient’s bed would reduce the need for additional cables or sensors could facilitate earlier transfer to a general ward, while enabling more comprehensive monitoring on general wards than is currently feasible. In intensive care, where continuous respiratory monitoring is critical for patients on mechanical ventilation or at risk of respiratory failure, a contactless system could reduce the need for adhesive sensors, lower the risk of skin irritation, minimize interference with other medical devices, and reduce personnel costs through simpler handling.

## Conclusion

Radar-based monitoring represents a promising, contactless, and non-intrusive approach for continuous recording and assessment of multiple respiratory metrics. It provides accurate and reliable data without imposing discomfort or restrictions on patients, unlike traditional methods. This study demonstrated that radar can not only estimate RR with high accuracy but also reliably capture metrics that are less commonly addressed in non-contact measurement research. While minor biases and increased variability were observed for TI, TE, and I:E ratio compared to RR, the overall agreement remained within clinically acceptable limits. These findings confirm the feasibility of extracting detailed temporal respiratory metrics from radar signals and highlight their potential for future applications in continuous, unobtrusive respiratory monitoring.

## Supplementary Information

Below is the link to the electronic supplementary material.

Supplementary Material 1

## Data Availability

The raw radar and reference respiratory data supporting this study are publicly available at figshare via the following direct link: [https://doi.org/10.6084/m9.figshare.12613109](https:/doi.org/10.6084/m9.figshare.12613109)^39^. The dataset is fully accessible and downloadable without restrictions. No additional raw data were generated in this study. Processed data and analysis code are available from the corresponding author upon reasonable request.
