# Supplementary material for: Radar-based inspiratory-to-expiratory time ratio estimation: a validation study
Source: Sci Rep. 2026 Mar 4;16:8256. doi: 10.1038/s41598-026-42517-9 (PMC12963625; doi:10.1038/s41598-026-42517-9)
Supplement: Supplementary file 1 — Supplementary Material 1 [file 41598_2026_42517_MOESM1_ESM.docx]

**Supplementary Information**

For: Radar-based Inspiratory-to-Expiratory Time Ratio Estimation: A Validation Study

**Supplementary Methods**

**Radar Signal Demodulation and Filtering**

Respiratory displacement was extracted from the complex in-phase and quadrature (I/Q) radar signals using ellipse reconstruction followed by arctangent demodulation. In an ideal continuous-wave radar system, the I/Q trajectory forms a circular locus centered at the origin when monitoring periodic target motion. In practice, hardware-related imperfections such as channel gain and phase imabalances distort this trajectory into an offset ellipse [34]. Ellipse reconstruction compensates for these non-idealities by estimating and removing the ellipse center and scaling the axes, thereby restoring a circular trajectory and ensuring that the derived phase reflects target displacement rather than system artifacts [45].

After ellipse reconstruction, the instantaneous phase was obtained via arctangent demodulation. As the arctangent function yields phase values wrapped to the interval $\left[ -\pi,\pi\right]$, phase unwrapping was applied to obtain a continuous phase signal representing thoracic motion.

To isolate the respiratory component, a fourth-order Butterworth bandpass filter with cutoff frequencies of 0.07–0.4 Hz was applied to the displacement signal. This frequency range corresponds to respiratory rates (RRs) between approximately 4–24 breaths per minute (brpm), encompassing normal resting respiration in healthy adults while allowing for moderate variability.

The same bandpass filter was applied to the reference signal derived from impedance pneumography to ensure consistent spectral content across modalities. As the radar and reference signals are expressed in different physical units (millimetres and Ohms, respectively), both filtered signals were normalized using a standard scaler prior to feature extraction.

**Respiratory Extrema Detection and Cycle Definition**

Respiratory cycles were identified by detecting local extrema in the filtered radar and reference signals. Local maxima, referred to as peaks (PKs), and local minima, referred to as valleys (VLs), were defined mathematically as follows:

- **Peak (PK)**: A point where the first derivative of the signal is zero (dx(t)/dt = 0), and the second derivative is negative (d²x(t)/dt² < 0).
- **Valley (VL)**: A point where the first derivative of the signal is zero (dx(t)/dt = 0), and the second derivative is positive (d²x(t)/dt² > 0).

A complete respiratory cycle was defined as a sequence consisting of an inspiratory phase from VL to PK, followed by an expiratory phase from PK to the subsequent VL.

Local extrema were identified by locating points in the respiratory signal that represent PKs and VLs under a set of predefined mathematical and physiological constraints. Specifically, extrema were required to exceed their surrounding signal values by a minimum relative amplitude (prominence), to be separated from adjacent extrema by a minimum temporal distance, and to exhibit a minimum temporal width. These criteria ensure that detected extrema correspond to physiologically plausible respiratory phase transitions rather than artifacts.

This procedure was implemented using the *find_peaks()* function from the *scipy* Python library (version 1.15.2), which provides a flexible framework for enforcing these criteria during extremum detection. Because real-world respiratory signals may contain artifacts or irregular fluctuations, additional constraints were imposed to exclude extrema that were unlikely to correspond to true physiological phase transitions.

**Artifact Rejection and Physiological Constraints**

To suppress artifact-related extrema, three physiologically motivated criteria were applied during peak detection:

- **Prominence**: Measures how much a PK stands out from neighbouring signal depressions. The minimum prominence threshold was set to 0.5 of the standard deviation of all candidate PKs within a recording.
- **Distance**: Refers to the minimum temporal distance between consecutive PKs. At a RR of 30 brpm, a full breath cycle lasts approximately 2 seconds, which serves as a lower bound for inter-peak distance.
- **Width**: Reflects the temporal extent of a PK at half prominence. Assuming a RR of 30 brpm and a conventional I:E ratio of 1:2, the expected TI is approximately 2/3 seconds, which is used as the minimum width threshold.

PKs not satisfying these criteria were excluded. VLs were detected by applying the same procedure to the inverted respiratory signal and mapping the resulting indices back to the original signal.

Because PKs and VLs were detected independently, their temporal sequence was constrained to follow the expected physiological order (VL → PK → VL). In cases where multiple extrema of the same type occurred consecutively (e.g., PK → PK or VL → VL), only one extremum was retained. Specifically, the first PK and the last VL within such a sequence were preserved. This strategy reflects the typical asymmetry between inspiratory and expiratory phases, with inspiration generally being shorter than expiration, and provides a pragmatic approach to handling noisy or irregular signals while maintaining physiologically plausible cycle definitions.

Once respiratory cycles were identified, cycles with a total duration exceeding 10 seconds (corresponding to a RR below 6 brpm) were classified as outliers and excluded from further analysis, as such values were considered physiologically implausible for healthy volunteers in a resting condition.

**Sliding Window Metric Computation**

Within each window, only complete cycles, defined as those where both the starting and ending VLs fall within the 2-minute window, were considered. For each respiratory cycle, TI was calculated as the time interval from VL to PK, and TE as the interval from PK to the subsequent VL.

To minimize the influence of occasional extreme values without discarding information from the majority of cycles, cycles with the smallest and largest TI and TE within each window were excluded prior to averaging. Depending on the number of available cycles, a minimum of two and a maximum of four cycles were removed.

After outlier exclusion, mean TI and mean TE are calculated from the remaining cycles. Mean RR was determined by taking the inverse of the total cycle duration (TI + TE)for each cycle and averaging across cycles within the window. Similarly, the mean I:E ratio was obtained by computing the ratio TI/TE for each cycle and averaging these ratios. Each sliding window was ultimately represented by a single set of mean respiratory metrics, which were used for subsequent statistical analyses.

**Supplementary Figures**

**
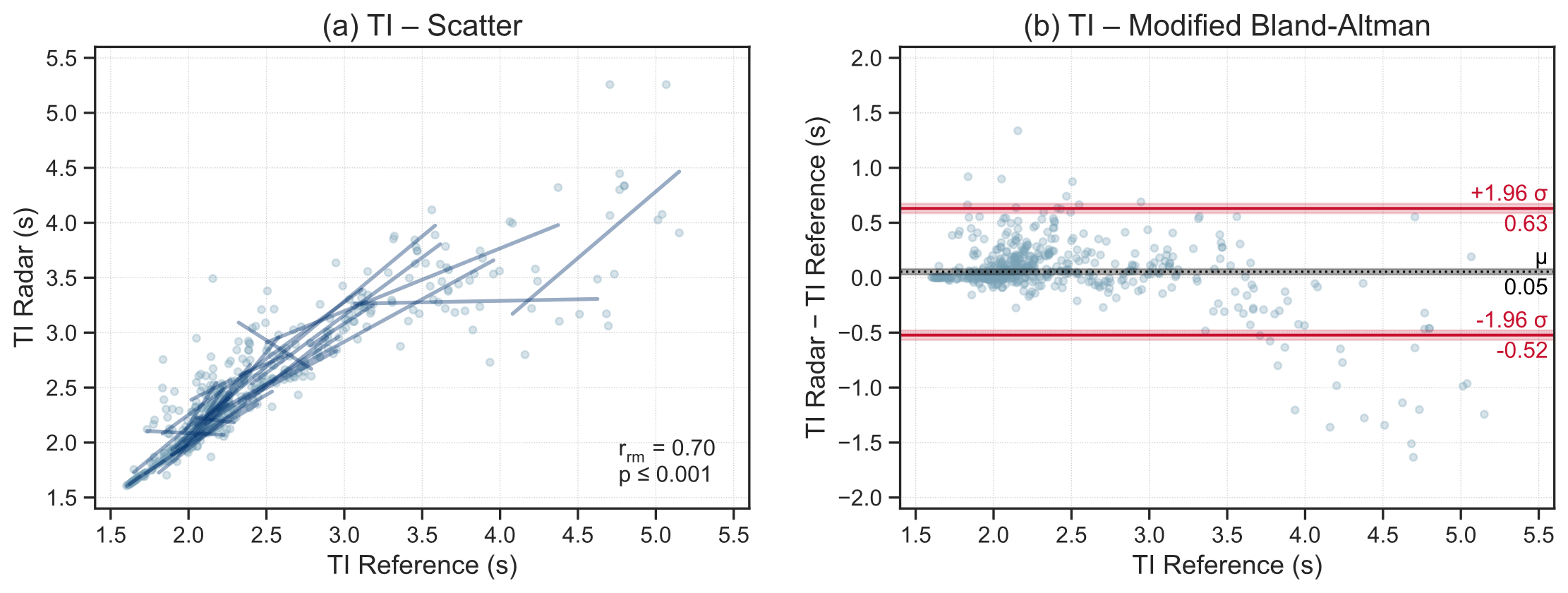
**

**Supplementary Fig. S1**. (a) Scatter plot and (b) modified Bland-Altman plot for radar- and reference-derived inspiratory times (TIs). Scatter plots show within-subject repeated-measures correlations r_rm_, and modified Bland-Altman plots display mean bias μ and limits of agreement (±1.96σ).


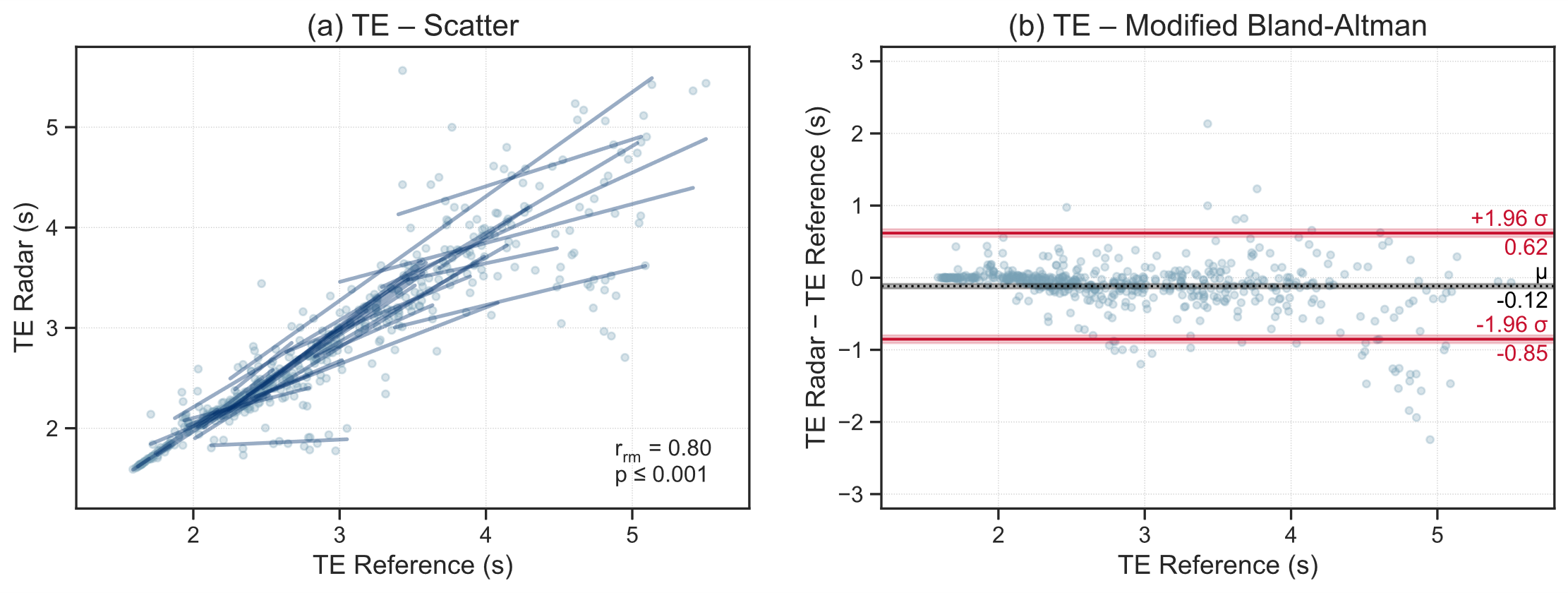


**Supplementary Fig. S2**. (a) Scatter plot and (b) modified Bland-Altman plot for radar- and reference-derived expiratory times (TEs). Scatter plots show within-subject repeated-measures correlations r_rm_, and modified Bland-Altman plots display mean bias μ and limits of agreement (±1.96σ).
